# Comparison of anti-malarial drug efficacy in the treatment of uncomplicated malaria in African children and adults using network meta-analysis

**DOI:** 10.1186/s12936-017-1963-0

**Published:** 2017-08-03

**Authors:** Solange Whegang Youdom, Rachida Tahar, Leonardo K. Basco

**Affiliations:** 1 University of Yaounde I, National Advanced School of Engineering, PO Box 8390, Yaounde, Cameroon; 2 Unité Mixte de Recherche 216 Mère et Enfant face aux Infections Tropicales (MERIT), Institut de Recherche pour le Développement (IRD), Université Paris Descartes, Laboratoire de Parasitologie, Faculté de Pharmacie, 4 avenue de l’Observatoire, 75006 Paris, France; 3 Unité de Recherche sur les Maladies Infectieuses et Tropicales Emergentes (URMITE), Aix Marseille Université, UM 63, CNRS 7278, IRD 198, INSERM 1095, Institut Hospitalo-Universitaire (IHU), Méditerranée Infection, 19-21 boulevard Jean Moulin, 13385 Marseille, France

**Keywords:** *Plasmodium falciparum*, Anti-malarial drug, Drug resistance, Clinical efficacy, Bayesian modelling, Multiple treatment meta-analysis

## Abstract

**Background:**

Artemisinin-based combination therapy (ACT) and novel drug combinations are available and used in African countries to treat uncomplicated malaria. Network meta-analysis methods are rarely and poorly applied for the comparison of their efficacies. This method was applied on a set of randomized controlled trials to illustrate its usefulness.

**Methods:**

A literature review available in Pubmed was conducted in July 2016. Eligible studies, conducted in sub-Saharan Africa, published between 2002 and 2016, focused on randomized controlled trials of at least two artemisinin-based combinations to treat uncomplicated malaria in children and adults. Agglomerate data were: the number of PCR-corrected adequate clinical and parasitological response (ACPR) on day 28, used as the primary endpoint in all interventions, the number of participants and the list of treatments. A Bayesian random effect meta-analysis using a binary outcome was the method to compare the efficacy. Ranking measure was used to obtain a hierarchy of the competing interventions.

**Results:**

In total, 76 articles were included; 13 treatment regimens were involved and tested in 36,001 patients. Using artemether–lumefantrine (AL) as the common comparator for the entire network, 12 relative treatment effects were estimated and indirect comparisons were obtained. Dihydroartemisinin–piperaquine (DHAP) was shown to be more effective than AL (odds ratio [OR] = 1.92; 95% CI 1.30–2.82; 19,163 patients), ASAQ (OR = 1.70; 95% CI 1.10–2.64; 14,433 patients), and amodiaquine–sulfadoxine–pyrimethamine (AQSP): OR = 2.20; 95% CI 1.21–3.96; 8863 patients. Artesunate–amodiaquine (ASAQ) was comparable to AL (OR = 1.11; 95% CI 0.84–1.45; 21,235 patients). No significant difference was found between artesunate and mefloquine (ASMQ) and AL (OR = 1.20; 95% CI = 0.52-2.8; 13,824 participants). According to treatment ranking, among the WHO-recommended ACT medicines, DHAP was shown to be the most efficacious.

**Conclusions:**

Based on the available evidence, this study demonstrated the superiority of DHAP among currently recommended artemisinin-based combinations. The application of the methods described here may be helpful to gain better understanding of treatment efficacy and improve future decisions. However, more data are needed to allow robust conclusions about the results in comparison with novel drugs. Further surveillance of the efficacy of anti-malarial drugs and clinical trials are needed to closely follow the evolution of the epidemiology of drug-resistant malaria in Africa.

**Electronic supplementary material:**

The online version of this article (doi:10.1186/s12936-017-1963-0) contains supplementary material, which is available to authorized users.

## Background

Artemisinin-based combination therapy (ACT) is currently the treatment of choice for uncomplicated *Plasmodium falciparum* malaria in Africa and elsewhere in the world. Since 2006, four ACT medicines have been recommended by the World Health Organization (WHO): artemether–lumefantrine (AL), artesunate–amodiaquine (ASAQ), artesunate–mefloquine (ASMQ) and artesunate–sulfadoxine–pyrimethamine (ASSP) [1, 2]. More recently, dihydroartemisinin–piperaquine (DHAP) has been added to the list of WHO-recommended ACT medicines [3]. To date, more than 80 countries worldwide have adopted ACT as the first-line therapy. Each of these five ACT medicines has several advantages and disadvantages, including safety, tolerability, dosing, post-treatment prophylactic effect, resistance to the partner drug, and price. To addition to these artemisinin-based combinations, a non-ACT combination amodiaquine–sulfadoxine–pyrimethamine (AQSP), had been employed for treatment in African countries, especially during the transition period from chloroquine or sulfadoxine–pyrimethamine monotherapy to ACT in the early 2000s.

Although artemisinin-resistant *P. falciparum* has emerged and is spreading in Southeast Asia [4, 5], as of 2017, Africa still seems to be spared. In the clinical protocol standardized by the WHO, the following classification with four categories is used to assess the parasitological and clinical outcome [6, 7]: adequate clinical and parasitological response (ACPR), late parasitological failure, late clinical failure, and early treatment failure.

Based on this classification, several individual studies and traditional meta-analyses have been carried out to assess the efficacy of different ACT medicines and alternative drugs in Africa [8–13]. A large majority of these studies have been conducted in children less than 5 years old, who bear the brunt of symptomatic malaria in areas of intense transmission. The traditional meta-analysis assumes identical two treatment arms in all randomized trials and is based on head-to-head comparison. However, more complex situations occur when pooling studies with either more than two treatment arms or no identical treatment arms between studies. Accordingly, network meta-analysis (NMA) may offer an enormous potential for a novel methodological approach to meta-analysis.

NMA, also known as mixed treatment comparison (MTC), is a statistical method to combine data from randomized comparisons A versus B, A versus C, B versus D, etc., to generate an internally consistent set of estimates while respecting the randomization in the evidence [14]. An increasing number of systematic reviews use NMA to compare three or more treatments to each other even if they have never been compared directly in a clinical trial [15–18]. Contrary to the traditional meta-analysis that estimates a common effect of the same intervention A and B among studies, MTC provides estimates of the effect of each intervention relative to each other. Therefore, NMA was designed only for randomized controlled trials and provides the ability to increase precision of point estimate and draw inferences on the comparability between interventions that have never been compared in a clinical trial. The probability that estimates which treatment is the most effective can also be calculated [19, 20] using fixed or random effect models. Recently, a new technique was developed to rank the competitive interventions according to their efficacy and safety [19–21]. The NMA method can be applied to both binary and continuous data (aggregate data and individual patient data) and could be extended to longitudinal data with various types of outcomes. Although individual patient meta-analysis is the current gold standard for evidence synthesis, analysis using individual data should consider the hierarchy of different interventions.

To date, only one study performed MTC of the WHO-recommended ACT using the WHO categorical outcome [22]. However, this work was limited to data from only one country (Cameroon) without treatment ranking assessment. Although Donegan et al. [23] have reviewed methods to assess different assumptions in NMA and applied the method to one existing trial of four ACT medicines, there is a need to extend the method to other anti-malarials. In another hand, studies published by the Worldwide antimalarial resistance network (WWARN) group were based on trials that included both one treatment arm and at least two treatment arms [12]. The authors compared drugs in terms of efficacy and prophylactic effect on individual patient data. However, the study did not present the network component of trials and also did not address the following questions: which drug combination is the most effective based on existing clinical data and what is the order of their relative efficacies in Africa? At present little is known about the efficacy of anti-malarials using NMA methods.

In the present work, two models with different assumptions proposed in the articles of NMA with binary outcome were assessed. NMA methods were applied on aggregate data from randomized trials to provide a full comparative efficacy of treatments.

## Methods

### Search strategy and outcome

Published literature was searched in PubMed. The search included all potentially relevant published articles, characterized by random allocation to treatment and comparison of different artemisinin-based combinations in Africa, starting from the year of the introduction of ACT (2002–2003), up to June 2016. An electronic search of Medline was conducted using the following key words: (“malaria” AND ACT [All Fields]) AND (Randomized Controlled Trial [ptyp]) AND (“2002/01/01” [PDAT]: “2016/06/30” [PDAT]) AND “humans”. In addition, reference lists of reviews were also screened to include potential articles. Studies were eligible if they involved at least 2 ACT medicines and reported clinical efficacy as ACPR corrected by polymerase chain reaction (PCR). Cure, i.e. the term “adequate clinical and parasitological response”, was defined as undetectable parasitaemia with or without fever, without previously meeting the criteria of treatment failure on the last day of follow-up (usually day 28).

### Selected articles and data

**Fig. 1:**
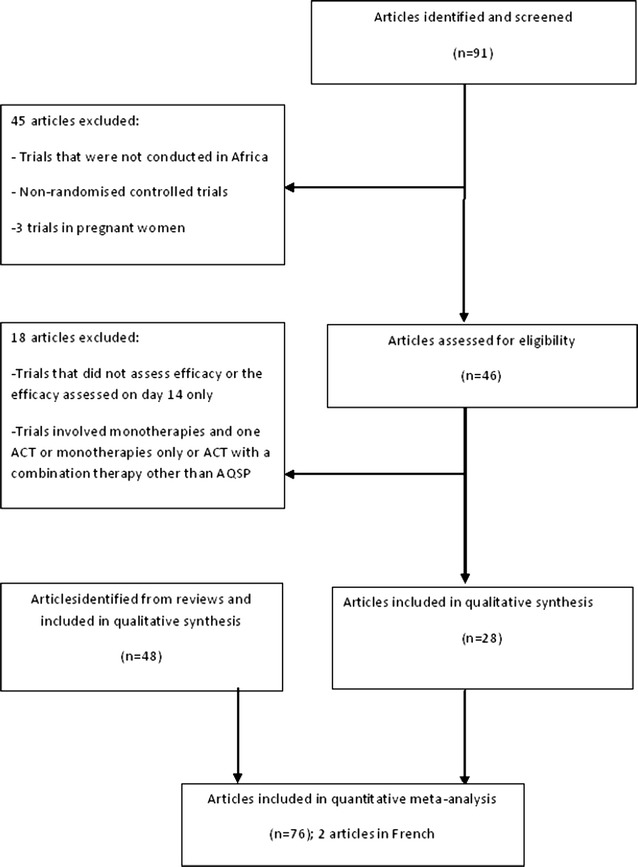
Flow chart diagram for the selected articles

A total of 91 articles was identified and screened. Details are presented in Fig. 1. Several articles were excluded for the following reasons: studies that were either on tolerability or recurrence of parasitaemia or did not report data on ACPR [24, 25], or based on the mechanism of resistance to ACT [26], or comprised only one ACT-arm as in Ref. [27], which compared AL to azithromycin–chloroquine (AZCQ). Data comprising monotherapies and ACT were excluded. In addition, reference lists in 16 reviews were screened for recent studies [12, 13, 22, 28, 29]. At the end of the screening process, 76 articles were included in the original analysis; *K* = 13 treatments were involved. The quinine arm was removed from a 3-arm study [30], and two studies published in French were included [31, 32]. The list of the interventions was artesunate–amodiaquine (ASAQ), artesunate–sulfadoxine–pyrimethamine (ASSP), artesunate–sulfamethoxypyrazine–pyrimethamine (ASSMP), artemether–lumefantrine (AL), dihydroartemisinin–piperaquine (DHAP), dihydroartemisinin–piperaquine–trimethoprim (DHAPT), artesunate–chlorproguanil–dapsone (ASCD), ASMQ, artesunate–atovaquone–proguanil (ASATPG), artemisinin–naphtoquine (ASNAPH), artesunate–amodiaquine–chlorpheniramine (ASAQCPH), artesunate–pyronaridine (ASPY) and, the non-ACT combination amodiaquine–sulfadoxine–pyrimethamine (AQSP). The combination AQSP was included in the present analysis because this combination was employed during the transition period before the adoption of ACT in many African countries and its efficacy had been compared to that of ACT in randomized studies.

Patient populations included children and adults: 82% were children less than 15 years old among whom more than 80% were children under 5 years. Some published articles involved both children and adults or adults only. Trial characteristics are described in the Additional file 1. Most of the studies presented the outcome on day 28 only, while few followed the patient until day 42 or even day 63. Hence, only a few studies had longitudinal data on days 28, 42 and 63. Accordingly, the obtained information was pooled on the primary end-point day 28 using the observed number of ACPR. The percentage of ACPR was estimated using the number of enrolled population or the number followed as far as the intention-to-treat (ITT) analysis or a per protocol (PP) approach is concerned, i.e. when the ITT results were not reported, the percentage of ACPR obtained in a PP approach was used to estimate the number of patients with positive outcome.

### Configuration of the anti-malarial network and drug numbering

**Fig. 2:**
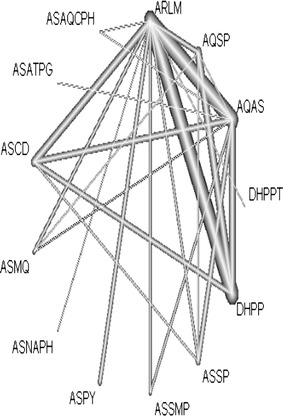
Malaria evidence network. Network of malaria treatment constructed from 76 studies with 13 therapies. The thickness of the line is proportional to the number of randomised clinical trials that have been included. The lines AL–DHAP and AL–ASAQ are the thickest. AL is the most tested ACT. No study directly compared AL and ASATPG but the estimate was obtained from indirect comparison using AL–ASAQ and ASAQ–ASATPG trials. This Figure was generated using the netmeta package (version 0.8-0) available in the statistical software R downloadable at http://cran.r-project.org

A network of treatments was built after extracting information from the articles. Figure 2 shows the set of nodes represented by the name of ACT or AQSP, linked by lines. The thickness of the line is proportional to the number of randomized clinical trials that have been included. The lines joining AL-DHAP and AL-ASAQ are the thickest. The Figure has a complex structure and allows indirect comparison, e.g. ASMQ and ASSMP with ASAQ as the reference. Close loops like AQSP, ASAQ and ASMQ provide both direct and indirect evidence. The network also shows that AL was the most studied ACT. It could be used as the comparator treatment. However, ASAQ was the most commonly used ACT in clinical practice in Africa due to its cost, availability and tolerability. It has been shown in several randomized clinical trials that ASAQ is comparable to AL. Accordingly, AL was used as the entire network common comparator among trials as it has been evaluated against the highest number of treatment. Therefore, the network trial generated *K* – 1 = 12 contrasts representing the overall relative treatment difference to be estimated from the *K* (*K* − 1)/2 = 78 direct and indirect overall comparisons.

The numbers of multi-arms studies were as follows: 2-arm (64 studies), 3-arm (9 studies), and 4-arm (3 studies). The numbers of studies per-treatment arm were as follows: 12 AQSP, 63 AL, 42 ASAQ, 1 ASAQCPH, 1 ASATPG, 4 ASCD, 6 ASMQ, 2 ASNAPH, 2 ASPY, 4 ASSMP, 11 ASSP, 22 DHAP, and 1 DHAPT. A detailed summary regarding the number of studies per-treatment arm, randomized sample sizes, number evaluated for the outcome, and proportion of ACPR is given in Table 1 for each treatment. For example, AQSP was found in 12 articles but only 7 compared its efficacy to that of AL. All cure rates ranged from 64.6 to 98%.

**Table 1:** Description of the data set in terms of studies per treatment and the sample sizes, on day 28

Treatments	Studies per treatment arm	Studies tested the trt with AL	Total sample size	Nbr of ACPR	% of ACPR
AL	63	–	12,981	11,426	88
AQSP	12	7	2681	1731	64.56
ASAQ	42	31	8254	7402	89.6
ASAQCPH	1	1	54	47	87.03
ASATPG	1	0^a^	100	95	95
ASCD	4	4	1591	1326	83.3
ASMQ	6	5	843	800	94.89
ASNAPH	2	2	147	144	97.95
ASPY	2	2	1204	1105	91.77
ASSMP	4	3	1545	1461	94.5
ASSP	11	3	1752	1593	91
DHAP	22	18	6182	4969	80.3
DHAPT	1	1	212	204	96.2

Data were extracted for all studies. The combination AL was the most frequently evaluated drug combination within the data. Few studies tested novel combinations. There was no study comparing ASATPG directly to AL

*Nbr* number, *trt* treatment, *ACPR* adequate clinical and parasitological response corrected by PCR

Based on the information obtained from the data set, the drugs were numbered as follows except for AL, i.e. AL is treatment number 1 representing the common comparator drug, 2 = AQSP, 3 = ASAQ, 4 = ASAQCPH, 5 = ASATPG, 6 = ASCD, 7 = ASMQ, 8 = ASNAPH, 9 = ASPY, 10 = ASSMP, 11 = ASSP, 12 = DHAP, and 13 = DHAPT. For each study, data were summarized in terms of the number of treatment arms, list of treatments, number of PCR-corrected day 28 ACPR in each treatment arm, and sample size in each arm.

### Statistical methods

In order to simultaneously compare all treatments in a coherent manner, NMA was used to obtain a hierarchy of the competing interventions [15]. This method required assumptions, notations and presentation of the modelling approaches.

### Assumptions

The application of NMA to the selected data assumed that the randomization process was preserved within each trial comparing the estimates of relative effect among treatments. Different assumptions were considered in NMA: (i) the hypothesis of homogeneity, i.e. no variation in treatment effect between trials within pairwise contrast; (ii) the consistency assumption establishing that within a closed loop, estimates for a particular pairwise contrast from head-to-head evidence is the same as what is estimated from the indirect evidence, and (iii) the inconsistency assumption, which means that estimates for a direct evidence are different from the indirect evidence.

For the following, the modelling approaches presented in a technical support document conceived by Dias et al. [33], was used. It is based on a random effect model with homogeneous variance, which allows assessing the validity of the consistency assumption and adjustment for multiple-arm trials. Notations described by Greco and Hong [34, 35] were followed to perform NMA for binary outcome using a Bayesian approach on aggregate data. The principal summary measure was the odds ratio (OR).

### Notations

Let *N* = 76 and *K* = 13 denote the number of randomized clinical trials and the total number of treatments, respectively. Given that each study *i* included *na*_*i*_ number of treatment arms, *i* = 1,…, *N*, and the number of PCR-corrected *ACPR r*_*i,k*_ in every arm *k* (*k* = *1, …, na*_*i*_ ≤ *K*) of study *i*, then *r*_*i,k*_ follows a binomial distribution denoted ~*Binom (p*_*i,k,*_ *n*_*i,k)*_, where *n*_*i,k*_ is the sample size of arm *k* in study *i*, and *p*_*i,k*_ is the probability of an event (success, here *ACPR*) of arm *k* in study *i*. For *K* and *N*, the number of existing comparisons (edges) between the treatments is equal to 109. This number was obtained by cumulating the number of pairwise comparisons among trials, i.e. the *na*_*i*_ *(na*_*i*_ − *1)/2* comparisons. For instance, given a comparator within a trial, a 2-arm trial contributes one pairwise comparison, a 3-arm trial, three comparisons and a 4-arm trial, six possible comparisons. Given the *K* = 13 treatments, *K* (*K* − 1)/2, i.e. 78 treatment effects, are expected. The baseline (also called basic parameters) *K* – 1 = 12 has to be estimated. Let the following $d_{12} , d_{13} , d_{14} , d_{35} , d_{16} , d_{17} , d_{18} , d_{19} , d_{1,10} , d_{1,13}$, $d_{1,12} , d_{1,13}$ denote the baseline parameters, representing the overall treatment effect, with *d*_*35*_ referring to the direct effect between ASAQ and ASATPG. The probability of event occurrence *p*_*ik*_ is modelled on the logit scale as:

1 $$\theta _{{ik}} = logit\left( {p_{{ik}} } \right) = \left\{ {\begin{array}{*{20}l} {\mu _{i} ;i = 1,2, \ldots ,N;k = b = 1,2, \ldots ,K} \\ {\mu _{i} + \delta _{{i,bik}} ;i = 1,2, \ldots ,N;k > b;k = 2,3, \ldots ,K} \\ \end{array} } \right.$$

where $\mu_{i}$ is the “study” random effect that accounted for differences among trials. This is also the trial-specific baseline and represents the log-odds ratio (LnOR) of event in the overall control treatment, while $\delta_{i,bik}$ is the trial-specific LnOR of event occurrence of the treatment *k* compared with the “placebo” treatment *b*_*i*_, which can be considered here as a random variable that follows a normal distribution *N (d*_*bk,*_ $\sigma_{i,bk}^{2}$ *)*, assuming that *b*_*i*_ = *b* for all studies. Therefore, treatment indexed *k* = *b* = 1 corresponds to AL, and *d*_*1,1*_ = *d*_*k,k*_ = *0*. In the context of NMA, it is assumed that the study-specific treatment effects are exchangeable such that $\sigma_{i,bk}^{2}$ = *τ* ^*2*^, ∀ *b*,*k*; i.e. τ^2^, the between studies variation, is assumed to be the same for all pairwise contrasts in all subsequent methods. $\delta_{i,bik} \sim N\left( {d_{bk} ,\tau^{2} } \right)$ is seen as the common form of a random effect meta-analysis, especially in the frequentist methods. Here, the parameter $\tau^{2}$ explains the variability among trials that could be caused by different drug formulations, the methods used, study designs, and the level of transmission that varies from one trial to another. In addition, the node $\sigma_{i,bk}^{2}$ expresses the same variability within a study among a pairwise contrast.

To account for heterogeneity in the patient populations, a dummy variable called *S*_*i*_ was defined as 1 if the population was children less than 15 years and 0 in other patient populations. A random coefficient β_i_ for *S*_*i*_ was assumed. Hence, β_i_ followed a normal distribution with mean β_0_ and variance ε^2^.

### Modelling approach 1: consistency

The model is given by:

2 $$logit\left( {p_{i,k} } \right) = \mu_{i} + \delta_{i,bk} + \beta_{i} S_{i}$$

where the node $\delta_{i,bk}$ is a random effect with mean *d*_*bk*_ representing the treatment effect of *b* compared to *k* for all studies (*k* ≠ *b*) and a variance $\sigma_{i,bk}^{2}$. This model provided the estimates of the *K* − 1 = 12 “basic parameters” representing all treatment relative to treatment *b*, by considering *b* = 1, 3. The parameter $\sigma_{i,bk}^{2}$ can be expressed in terms of the study variation τ and the number of “basic parameters”, i.e. $\sigma _{{i,bk}}^{2} = \tau ^{2} \times {{2\left( {K - 1} \right)} /K}$. For purpose of illustration, to derive the treatment difference between 2 = AQSP and 4 = ASAQCPH given AL as the control group, the consistency equation is given by *d*_*24*_ = *d*_*14*_ − *d*_*12*_.

### Modelling approach 2: inconsistency

While heterogeneity is characterized as between-trial variation within treatment contrast, inconsistency is the variation between contrasts. Contrary to the consistency model, there is no common comparator across studies. Several methods have been studied to test the inconsistency assumption and are considered as an extension of Bucher’s method [36]. Instead of the repeat application of the method, Dias et al. [33] proposed to compare the consistency model with an inconsistency model. Hence, for a study comparing treatment *k* to treatment *k* ^*’*^, the model was defined as follows:

3 $$logit\left( {p_{i,k} } \right) = \mu_{i} + \delta_{{i,kk^{\prime}}} + \beta_{i} S_{i}$$

In this model, within study *i*, each node $\delta_{{i,kk^{\prime}}}$ is a random variable with a mean treatment effect $d_{{kk^{\prime}}}$ with a variance of $\sigma_{i,bk}^{2}$. $\delta_{{i,kk^{\prime}}}$ was treated as a separate parameter to be estimated. Accordingly, the model gives estimates of 12 overall treatment effects and also provides estimates of other 66 treatment effects. To test if consistency is reasonable, model 3 was compared to model 2 by checking statistical difference of their model fit.

### Adjustment for multiple arms studies

Since there are 2-, 3- and 4-arm trials, the difference in treatment effects may arise from different sources and can be inconsistent [33]. Although only a small proportion of studies was 3-arm (*n* = 9) or 4-arm (*n* = 4) trials, with model 2, an adjustment for multiple arms trials was made. Indirect comparisons accounted for the correlation between any two treatment contrasts in a multi-arm trial. This correlation is equal to 0.5 given the hypothesis that the study variation is the same among all pairwise contrasts.

### Statistical data analysis

First, pairwise random effect meta-analyses were conducted. The method was implemented with the meta-analysis package of the software R (*rmeta*) downloadable at https://cran.r-project.org/. Treatments involved were the five WHO-recommended ACT medicines: ASAQ, ASSP, AL, DHAP and ASMQ. Direct estimates were extracted from head-to-head trials using the odds ratios and 95% confidence interval (CI). The heterogeneity of variance for each pairwise comparison was estimated using the $I^{2}$ statistic.

Secondly, data were analysed by Bayesian method. Prior information was defined for all unknown parameters *d*_*bk*_ and $\tau^{2}$. The following prior distributions were considered: $\mu_{i} \sim N\left( {0, 0.0001} \right);\, d_{bk} \sim N(0, 0.0001)$. The inverse of the study variance was given a uniform distribution, i.e. $s^{2} = \frac{1}{{\tau^{2} }}\sim Uniform(0,2)$; β0 ∼ *N* (0,0.0001), and 1/ε^2^ also followed a uniform distribution *dunif* (0,2). One markov chain was run. After a burn-in of 100,000 iterations, posterior outputs were obtained from the last 50,000 iterations. Model fit statistics were obtained for each model, i.e. deviance information criterion (DIC), deviance ($\hat{D}$) which provides an idea of the model likelihood, and $p_{D }$ the number of parameters in the estimation process. The model with the smallest DIC is considered the best. Regarding treatment ranking, the probabilities that each treatment is the best, second best, and third best on Day 28, among 13 treatments, were calculated according to van Valkenhoef and Kuiper [21]. Treatment ranks were based on posterior probabilities. In each MCMC run, every treatment was ranked according to its estimated magnitude. The proportion of MCMC cycles in which the treatment *k* ranks first yielded the probability that such specific treatment is the best among all treatments. Analyses were carried under the WinBUGS software. The WinBugs codes that were used for NMA are available in the Additional file 2.

## Results

### Head-to-head meta-analyses

Thirty-one articles (15,695 participants) compared the efficacies of ASAQ (taken as reference) and AL. The summary measure for these studies showed that the efficacy of ASAQ was comparable to that of AL (OR = 0.96; 95% CI [0.79; 1.17]; p value = 0.682; τ^2^ = 0.138; *I* ^*2*^ = 54% [32.2%; 69.9%]). The efficacy of ASAQ (as reference) was compared to that of DHAP in six studies (4042 participants); ASAQ appeared to be less efficacious than DHAP but the result was not statistically significant (OR = 0.81; 95% CI [0.54; 1.22]; p value = 0.31; *I* ^*2*^ = 59.5% [0.5%; 83.5%]). Seven studies (2245 patients) compared ASAQ (reference) to ASSP. Results showed that both treatments were comparable (OR = 0.77; 95% CI [0.52; 1.14]; p value = 0.191; τ^2^ = 0.055; *I* ^*2*^ = 26.7% [0%; 68.2%]). Eighteen articles (100,000 participants) compared AL (taken as reference) to DHAP. The results showed that AL was statistically less efficacious than DHAP (OR = 0.52; 95% CI = [0.36; 0.77], τ^2^ = 0.518, p value = 0.0009; *I* ^*2*^ = 86.8% [80.6%; 91%]), but AL was as effective as ASMQ in five studies (2869 patients; OR = 0.90; 95% CI [0.56; 1.47], τ^2^ = 0, *I* ^*2*^ = 0% [0%; 73.4%]). Three studies (1104 patients) compared AL and ASSP, and the differences were not statistically significant (OR = 1.14; 95% CI [0.40; 3.22], *I* ^*2*^ = 36.4% [0%; 78.7%). Only one study compared ASAQ to ASMQ. No study compared ASMQ to DHAP or ASSP. In addition, no study compared ASSP to DHAP.

### Comparing AQSP and ACT

Four trials (1584 participants) compared ASAQ (reference) to AQSP and showed that ASAQ was more efficacious than AQSP, but the results were not significant (OR = 1.51; 95% CI = [0.80; 2.87]; p-value = 0.20; τ^2^ = 0.23, *I* ^*2*^ = 63.4% [0%; 87.7%]). AQSP (reference) was found less efficacious than AL in seven trials (2316 participants) but the result was not statistically significant (OR = 0.76; 95% CI [0.25; 2.30]; p value = 0.62; τ^2^ = 1.89, *I* ^*2*^ = 93.7% [89.5%; 96.3%]) [37–43]. The same conclusion was found in three trials comparing AQSP (ref) to DHAP (2518 patients; OR = 0.54; 95% CI [0.16; 2.03]; τ^2^ = 1.30, *I* ^*2*^ = 96.6% [93%; 98.4%]) [42, 44, 45]. Forest plots of some of these results are illustrated in Additional file 3.

### Relative treatment effects from NMA

Table 2 displays the overall treatment effects (given AL as the common comparator) estimated from two models using the Bayesian approach and their 95% credible interval (CrI). All models yielded similar results. The difference in DIC of models 3 and 2 was 1025.04–1024.07 = 0.97, suggesting little evidence for inconsistency. Hence, model 2 gave the best adjustment to the data set. A significant difference was found between DHAP and AL. For example, with model 2, DHAP was 1.92-fold more efficacious than AL (OR = 1.92; 95% CrI = 1.30–2.82; 63 vs 22 studies per-treatment arm; 19,163 participants). New drug combinations like ASSMP, ASATPG, ASPY, and ASAQCPH appeared more efficacious than AL, but the results were not statistically significant as indicated by the confidence intervals. For model 3, the other 78 − 12 = 66 comparisons can be found in Additional file 3.

**Table 2:** Comparison of Bayesian models; posterior distributions of odds ratios and 95% credible intervals (CrI)

	Basic parameters	Model 2		Model 3	
		Consistency		Inconsistency	
		Odds ratios	95% CrI	Odds ratios	95% CrI
AQSP	$d_{12}$	0.87	0.50–1.51	0.96	0.45–2.05
ASAQ	$d_{13}$	1.12	0.82–1.54	1.09	0.76–1.57
ASAQCPH	$d_{14}$	1.55	0.27–8.76	1.91	0.24–14.95
ASATPG*	$d_{35}$	3.82	0.49–29.9	3.39	0.41–28.13
ASCD	$d_{16}$	0.59	0.26–1.32	0.76	0.26–2.18
ASMQ	$d_{17}$	1.20	0.52–2.80	1.26	0.47–3.36
ASNAPH	$d_{18}$	0.85	0.06–11.78	0.82	0.06–11.77
ASPY	$d_{19}$	1.41	0.42–4.75	1.41	0.38–5.23
ASSMP	$d_{1,10}$	1.15	0.46–2.90	1.65	0.48–5.68
ASSP	$d_{1,11}$	1.36	0.73–2.55	0.99	0.25–3.89
DHAP**	$d_{1, 12}$	1.92	1.30–2.82	1.88	1.20–2.95
DHAPT	$$ \begin{array}{*{20}c} { } \\ { d} \\ \end{array}_{1,13} $$	1.48	0.22–10.09	1.47	0.19–11.03
$\tau^{2}$ (SD)		1.45 (0.30)		1.26 (0.27)	
$\sqrt {\tau^{2} }$ (SD)		0.84 (0.08)		0.90 (0.097)	
$\hat{D}$		732		728	
$p_{D}$		145		148	
DIC		1024.07		1025.04	

Results are extracted from the random effects model under consistency and inconsistency assumptions. $d_{1k}$ is the relative effect of treatment *k* compared to treatment 1, here AL

*SD* estimate of the standard deviation of the parameter, *σ* estimate of the variability in treatment effect between trials within pairwise contrasts, *τ* estimate of the between study variation, $\hat{D}$: deviance estimating likelihood, $p_{D}$ number of parameters in the model, *DIC* deviance information criterion (when it is small, it gives the best model)

^a^DHAP was superior to AL in all fitted models

^b^ASATPG was the only ACT compared directly to ASAQ; the difference of efficacy of these ACT medicines was not statistically significant

### Indirect comparisons

Indirect estimates were calculated from each model assuming the consistency equation (Table 3). The results showed the superiority of DHAP compared to ASCD (OR = 3.25, 95% CI 1.46–7.25), ASAQ (OR = 1.70; 95% CI 1.10–2.64), and AQSP (OR = 2.20; 95% CI 1.21–3.96). Indirect estimate of DHAP vs ASMQ yielded OR = 1.59 with 95% CI 0.62–4.02 showing no significant difference between the two drugs. The efficacy of DHAP was also not statistically different from that of ASPY (OR = 1.36; 95% CI 0.38–4.87). The combination ASATPG was 3.40-fold more efficacious than AL, albeit not statistically significant (95% CI 0.60–18.56).

**Table 3:** Indirect comparisons calculated from the consistency equation

Ref	Plac	Study per comparison	Parameters	Consistency OR	Model 2 95% CI	Inconsistency OR	Model 3 95% CI
ASMQ	AQSP	2	$d_{27} = d_{17} - d_{12}$	0.63	0.26–1.52	0.67	0.22–1.97
DHAP^a^	AQSP	3	$d_{2,12} = d_{1,12} - d_{12}$	2.20	1.21–3.96	1.96	0.89–4.30
ASSP	AQSP	3	$d_{2,11} = d_{1,11} - d_{12}$	1.55	0.76–3.21	1.03	0.26–4.15
ASAQCPH	ASAQ	1	$d_{34} = d_{14} - d_{13}$	1.38	0.26–7.41	1.75	0.23–8.69
ASMQ	ASAQ	1	$d_{37} = d_{17} - d_{13}$	1.07	0.47–2.44	1.15	0.44–3.00
ASSMP	ASAQ	2	$d_{3,10} = d_{1,10} - d_{13}$	1.03	0.42–2.51	1.51	0.46–5.01
ASCD	ASAQ	2	$d_{36} = d_{16} - d_{13}$	0.52	0.23–1.15	0.69	0.25–1.94
ASSP	ASAQ	7	$d_{3,11} = d_{1,11} - d_{13}$	1.21	0.64–2.27	0.90	0.24–3.41
AQSP	ASAQ	4	$d_{32} = d_{12} - d_{13}$	0.78	0.41–1.46	0.87	0.38–2.04
DHAP^a^	ASAQ	6	$d_{3,12} = d_{1,12} - d_{13}$	1.70	1.10–2.64	1.72	1.01–3.07
DHAP	ASCD	1	$d_{6,12} = d_{1,12} - d_{16}$	3.25	1.46–7.25	3.17	1.21–8.23
ASSP	ASCD	1	$d_{6,11} = d_{1,11} - d_{16}$	2.31	0.95–5.61	2.49	0.78–7.86
DHAP	ASMQ	0	$d_{7,12} = d_{1,12} - d_{17}$	1.59	0.62–4.02	1.45	0.50–4.38
DHAP	ASPY	0	$d_{9,12} = d_{1,12} - d_{19}$	1.36	0.38–4.87	1.33	0.33–5.35
ASATPG	AL	0	$d_{15} = d_{35} - d_{13}$	3.40	0.42–27.31	3.10	0.36–26.62

The third column is the observed number of trials for each comparison. 0 comparison means the trial does not exist in the data set but with A one can estimate the treatment difference. $d_{kl} = d_{bl} - d_{bk}$ is the consistency equation used to derive all indirect comparisons

*Ref* reference group, Plac denotes the comparator. *OR* odds ratio is the overall effect estimated from the entire network, *CI* confidence interval

^a^Means the differences are significant. There was not enough evidence to support the higher efficacy of DHAP compared to ASMQ and ASPY, and there were insufficient data to compare ASATPG to AL. For loops having at least 3 treatments, the variance between treatments effects accounted for the correlation (equals to 0.5) between any pairwise contrasts. DHAP was more efficacious than AQSP and ASAQ

### Rank probabilities

Model 2 was selected for calculating the rank probabilities. Table 4 displays the rank for each treatment and the corresponding rank probability. Data are shown for rank 1, rank 2, rank 3 and for the worst treatment (rank 13). ASATPG combination had the highest probability (0.53) at rank 1, followed by ASNAPH (0.11) and ASAQCPH (0.11). At rank 2, DHAP was the drug with the highest probability (0.25). At rank 3, the best was also DHAP (0.31). For rank 13, the worst treatment was ASCD (0.48).

**Table 4:** Posterior distributions of the ranking probability for each treatment

Treatment *k*													
Rank *b*	1	2	3	4	5	6	7	8	9	10	11	12	13
	AL	AQSP	ASAQ	ASAQCPH	ASATPG	ASCD	ASMQ	ASNAPH	ASPY	ASSMP	ASSP	DHAP	DHAPT
1	0	0.004	0	0.11	0.53	0	0.01	0.11	0.037	0.006	0.005	0.065	0.10
2	0	0.02		0.15	0.16	6 × 10^−5^	0.03	0.09	0.09	0.023	0.026	0.25	0.14
3	10^−5^	0.06	0.001	0.10	0.07	3 × 10^−4^	0.069	0.054	0.10	0.004	0.068	0.31	0.09
4	8 × 10^−4^	0.11	0.008	0.08	0.05	9 × 10^−4^	0.10	0.04	0.11	0.06	0.12	0.22	0.0076
5	0.004	0.15	0.03	0.07	0.036	0.002	0.12	0.04	0.11	0.086	0.16	0.095	0.068
6	0.02	0.166	0.08	0.06	0.02	0.004	0.13	0.036	0.098	0.097	0.17	0.03	0.058
7	0.06	0.15	0.16	0.054	0.021	0.007	0.12	0.03	0.08	0.10	0.14	0.009	0.05
8	0.14	0.11	0.22	0.04	0.016	0.010	0.098	0.028	0.07	0.09	0.109	0.002	0.04
9	0.23	0.08	0.22	0.04	0.014	0.016	0.08	0.02	0.06	0.096	0.07	3 × 10^−4^	0.04
10	0.27	0.06	0.16	0.05	0.01	0.03	0.08	0.03	0.06	0.10	0.05	4 × 10^−4^	0.053
11	0.19	0.04	0.08	0.07	0.01	0.10	0.08	0.06	0.07	0.13	0.03	10^−6^	0.08
12	0.06	0.02	0.02	0.08	0.01	0.33	0.05	0.12	0.058	0.10	0.01	10^−6^	0.1
13	0.003	0.003	0.001	0.053	0.007	0.48	0.01	0.30	0.02	0.03	0.001	0	0.076

Results from model 2 are shown

Lines of the table are rank probabilities denoted from 1 (best) to 13 (worst). In each line, rank probability (rank *b*, *b* = *1,…, 13*) is given for each treatment, with ASATPG (0.53), followed by ASNAPH (0.11) and ASAQCPH (0.11). At lines 2 and 3, DHAP has the highest rank probability. At line 13, the highest probability 0.48 was obtained with ASCD

### Results among WHO-recommended ACT and AQSP

To provide an answer to the best treatment among WHO-recommended ACT medicines and AQSP, a second analysis was carried out using model 2, with AL as the overall control group. Treatments were numbered as follows: AL = 1, AQSP = 2, ASAQ = 3, ASMQ = 4, ASSP = 5, DHAP = 6, 7 = ASAQCPH, 8 = ASATPG, 9 = ASCD, 10 = ASNAPH, 11 = ASPY, 12 = ASSMP, and 13 = DHAPT, allowing the WHO-recommended ACT medicines to be the first 6 treatments. Ranking was only up to 6. Table 5 displays the results. DHAP was more efficacious than AL (OR = 2.09; 95% CI [1.54–2.83]). At ranks 1, 2, and 3, DHAP emerged at the first treatment rank.

**Table 5:** Results for AQSP and the WHO-recommended ACT medicines

Rank probabilities					
Treatments	Odds ratios	95% CrI	Rank 1	Rank 2	Rank 3
AL	–	–	0	10^−5^	10^−4^
AQSP	0.87	0.50–1.51	0.0004	10^−4^	0.005
ASAQ	1.12	0.82–1.54	0.00	10^−4^	0.007
ASMQ	1.20	0.52–2.80	0.0016	0.05	0.08
ASSP	1.35	0.71–2.58	0.01	0.05	0.10
DHAP	1.92	1.29–2.83	0.04	0.20	0.30

A total of 76 studies were analysed using the random effect model 2 under the hypothesis of consistency. Treatments were numbered as the first six combinations. Posterior distributions of odds ratio and 95% credible intervals (CrI) were extracted. AL was the comparator. Posterior rank probabilities are given for ranks 1, 2 and 3. In this model, DHAP was shown to be the most efficacious among the combination therapies

## Discussion

NMA of randomized clinical trials is becoming a promising tool to analyse grouped data with multiple interventions. The use of this methodological approach was assessed for the evaluation of malaria treatment efficacy and the selection of the most efficacious drug. Several clinical trials were identified and assessed using the proportions of PCR-adjusted ACPR. The rationale of choosing 2002–2003 as the starting point was that it is considered as the early years of the introduction of ACT in some African countries and the year when the WHO protocol requiring a 28-day follow-up to guide clinical trials was updated [6]. When using this approach, an examination of the type of primary outcome is important because it determines the choice of the modelling approach. Treatment outcome can be categorical or continuous, or it may express as survival outcome. To apply NMA, the included studies should be randomized trials that compare at least two treatments. A single-arm trial cannot be included in the analysis. Robust results can be expected with NMA, depending on the choice of the common end-point of drug efficacy and assessment of heterogeneity. Furthermore, it has been argued that the chief advantage of random-effect meta-analysis is that heterogeneity is taken into account in the modelling. Hypothesis tests provide information about consistency and inconsistency of the network.

The results become more robust when a Bayesian estimation method is used. One of the strengths of this method is that, at a point of time when the efficacy of a drug is not established, the contribution of other trials could suggest its relative efficacy. In addition, pooling trial increases the sample size and allows the selection of the best treatment. This is an advantage of NMA approach that may facilitate the selection of the best treatment through rank probabilities and is, therefore, one feature of the novel method to guide decision making. Two models were examined in the present study, and both provided similar results.

The results did not show any difference in the efficacies of ASAQ and AL. AL was the first co-formulated fixed-combination ACT to become available, followed by ASAQ. The six-dose regimen of AL is highly effective and represents a challenging comparator for any new drug combination although the non-inferiority of ASAQ versus AL had been shown in an earlier multi-centric study [46]. Both ACT are still highly effective and well tolerated with no serious adverse event, supporting their continuous use for the treatment of uncomplicated *P. falciparum* malaria in Africa [47, 48]. However, in a few studies these two drugs were found to have cure rates lower than the critical threshold of 90% required by the WHO [49, 50]. After PCR correction, the cure rates were 77.8 and 84.1% for AL and ASAQ, respectively [49]. Since cases of ACT resistance have been reported in South-East Asia, the need to understand the mechanism of artemisinin resistance in *P. falciparum* has become a global research goal. Since their adoption in African countries, few data on the selective impact of ACT in the circulating parasite population are available. A recent study assessed the selective impact of the treatment with ASAQ and AL on *Pfcrt* and *Pfmdr1* alleles and found no association between the presence of *Pfcrt* and *Pfmdr1* alleles before treatment and at the time of treatment failure [26]. Hence, countries that rely on ASAQ and AL for the first-line treatment should continue to monitor their clinical efficacy and molecular markers associated with resistance to these drugs, including *kelch* 13 [51].

The results highlighted that DHAP is superior to AL, contrary to some published reports in which the overall efficacy of DHAP was found to be similar to that of AL in multi-centric studies in Africa [52, 53] and in individual randomized clinical trials [30, 43, 47, 54–57]. However, the result of the present study is in agreement with a recent systematic review in which it was found that DHAP reduces overall treatment failure compared to AL [10]. In addition, indirect comparison also showed that DHAP was more efficacious than ASAQ and ASCD. These results should be taken with precaution as piperaquine has a longer elimination half-life than most other drug partners of artemisinin derivatives.

Studies in Africa also demonstrated the efficacy and tolerability of ASMQ [58, 59]. However, ASMQ has not been adopted by African malaria control units due to the high efficacy of AL and ASAQ and a relatively high incidence of side effects, in particular vomiting, associated with mefloquine. On the other hand, studies that compared the efficacies of ASMQ and AL had been conducted, but there is no conclusive evidence and argument to propose ASMQ as a replacement of AL in Africa [58–62]. The present analysis detected no significant difference in the efficacy of these two ACT medicines.

Regarding ASSP, it has been efficacious and well tolerated as ASAQ and, has been used in some African countries as the first-line treatment [63]. Recent literature and the present analysis support the comparable efficacy of ASSP and AL. However, the use of SP combined with artesunate is a source of concern in many African countries where molecular studies have shown increasing prevalence of multiple mutations in dihydrofolate reductase (*dhfr*) and dihydropteroate synthase (*dhps*) genes and where SP is employed for intermittent preventive treatment in pregnancy and infancy and AQSP combination is employed for seasonal malaria chemoprevention [3].

In addition to five ACT medicines currently recommended by the WHO [3], several novel combinations have been evaluated in the African continent in recent years. The triple combination dihydroartemisinin–piperaquine–trimethoprim (DHAPT), is administered over 2 days rather than the WHO-recommended, standard 3-day ACT administration, and its efficacy was found to be similar to that of AL in a three-centre study [64]. ASAQCPH resulted in a better haematological recovery and higher cure rates, as compared to ASAQ and AL in Nigeria [65]. The combination ASATPG was highly effective in Cameroonian young children [66]. ASNAPH is available in Africa and is recommended for use as a single-dose regimen by the manufacturer [31, 67]. ASSMP was assessed in several African countries, and its efficacy was found similar to those of ASAQ and AL [43, 68, 69].

ASPY is a newly introduced form of ACT which may possibly be deployed together with primaquine to kill mature *P. falciparum* gametocytes in an effort to reduce transmission in areas where malaria elimination programme is being implemented [70, 71]. A meta-analysis highlighted its efficacy and safety and concluded that it could be an option for the first-line treatment [11]. In the present analysis, ASPY was found to be more efficacious than AL and less efficacious than DHAP, but the results were not statistically significant.

So far, only a few studies have presented results based on the suggested methodological changes. However, Donegan et al. [23] explored and reviewed methods assessing key assumptions of NMA. These authors applied the methods on data from only one multi-centric study [72] comparing DHAP, ASAQ, AL and ASCD. The authors did not draw conclusions regarding the results and did not present treatment according to their efficacy rank.

Among non-ACT drug combinations, AQSP had been evaluated and compared to ACT in some African countries during the pre-ACT period [73, 74]. In some studies, AQSP was as efficacious as ASSP. AQSP has also been found highly efficacious and well tolerated as DHAP [45]. The results of the present study found no significant difference between AQSP and AL and between DHAP and AQSP. At present, AQSP cannot be recommended for the first-line treatment of uncomplicated malaria. However, the results presented in this study suggest the continued usefulness of AQSP in Africa, in particular for seasonal malaria chemoprevention.

Based on this novel methodological approach, among the 13 combination therapies included in the present analysis, ASATPG emerged at the top rank as the best treatment followed by ASAQCPH and ASNAPH in spite of the limited number of trials. These findings show that NMA tends to be too sensitive when treatments are tested once or twice. The method allowed these treatments to be at first rank. However, to counter this methodological limitation, further randomized clinical trials with AL, ASAQ, AQNAPH and ASATPG would be necessary to confirm these initial findings based on a limited number of trials.

The second analysis with the most widely tested combinations in Africa showed that DHAP was more effective than AL and found to be the best treatment, followed by ASMQ, and AL at the last rank. The combination AL has been found in most studies to be the most efficacious treatment. In the present analysis, the finding that AL is not the most efficacious ACT could be due to the fact that it was fixed as the overall control drug in the network. However, this result does not bring into question its efficacy because in the present analysis it was ranked among the best treatments. As an additional option, ASMQ, which has not been extensively employed in Africa, could be used as an alternative for the first-line anti-malarial therapy.

The present study provides the first overview of NMA methods for anti-malarial drug efficacy. It follows the work initiated by Donegan et al. and offers further research opportunities [23]. However, the study has some limitations. First, search criteria included studies conducted only in Africa. Secondly, although the method can be extended to individual patient data, the present study took into consideration aggregate patient data, which may have reduced the power of the study. However, results were similar with those obtained with individual outcome. Third, an adequate number of trials was not available for a robust inference for the recently tested new combination therapies. Despite this shortcoming, the findings described in this work reflect the reality in the field. Moreover, regarding the change in efficacy, inadequate dataset was available on day 42, day 35 and day 63 to perform separate analyses. Fourth, drug efficacy was limited to the outcome on day 28, as recommended by the WHO. According to WWARN [12], there is variability in efficacy related to drug formulation. As this variability was not known in the past, it was assumed in the present study that drug efficacy does not vary with formulation. The analyses included studies on both children and adults, and the majority of the included patients were children less than 5 years of age. This was done partly to include novel drugs and estimate their contributions to the anti-malarial efficacy. Accordingly, this approach may have increased heterogeneity and partly explain imprecise results. In addition, confounding factors such as age and doses may reduce the demonstrated effect. Nevertheless, the random effect model accounted for this heterogeneity by assuming a trial-random effect and a random coefficient for the type of study population. Both of these factors were introduced in the model to reduce the degree of heterogeneity to about 50%.

## Conclusions

NMA technique may have a role to play in the evaluation of different public health policies and interventions for malaria control. DHAP was found the best treatment overall but this observation should be treated with caution in decision-making since the present analysis was based on the outcome on day 28 and did not take into consideration the outcome on day 42 and 63 for drugs with long elimination half-life due to inadequate available data. More comparative studies are needed with novel drug combinations to assess their efficacy as compared to the currently recommended artemisinin-based combinations in Africa.

## Additional files

**Additional file 1.** Description of the trials characteristics. The file contains the description within each article in terms of trial characteristics, including the number of treatment arms (a), the list of treatment (k), the enrolled population (n) and the number of observed PCR-corrected ACPR (r). The file also contains the number of studies for each pairwise comparison.

**Additional file 2.** WinBUGS codes for the random effect models. The file contains two parts. Part 1 is the code to run the random effect model under the hypothesis of consistency; part 2 is the code for the random effect model under the inconsistency assumption. These codes are to be run using the free WinBUGS 1.4 software downloadable at http://www.mrc-bsu.cam.ac.uk/bugs/winbugs/contents.shtml.

**Additional file 3.** Forest plots and other direct and indirect comparisons. Direct and indirect comparisons are extracted from the model with inconsistency.

## Abbreviations

ACT: artemisinin-based combination therapy
ACPR: adequate clinical and parasitological response
AQSP: amodiaquine–sulfadoxine–pyrimethamine
AL: artemether–lumefantrine
ASAQ: artesunate–amodiaquine
ASAQCPH: artesunate–amodiaquine–chlorpheniramine
ASATPG: artesunate–atovaquone–proguanil
ASCD: artesunate–chlorproguanil–dapsone
ASMQ: artesunate–mefloquine
ASNAPH: artemisinin–naphtoquine
ASPY: artesunate–pyronaridine
ASSMP: artesunate–sulfamethoxypyrazine–pyrimethamine
ASSP: artesunate–sulfadoxine–pyrimethamine
AZCQ: azithromycin–chloroquine
CI: confidence interval
DHAP: dihydroartemisinin–piperaquine
DHAPT: dihydroartemisinin–piperaquine–trimethoprim
DIC: deviance information criterion
ITT: intention to treat
LnOR: log-odds ratio
MTC: mixed treatment comparison
NMA: network-meta-analysis
OR: odds ratio
PCR: polymerase chain reaction
PP: per protocol
SP: sulfadoxine–pyrimethamine
WHO: World Health Organization
WWARN: Worldwide antimalarial resistance network
