# Supplementary material for: Comparison of anti-malarial drug efficacy in the treatment of uncomplicated malaria in African children and adults using network meta-analysis
Source: Malar J. 2017 Aug 3;16:311. doi: 10.1186/s12936-017-1963-0 (PMC5543626; doi:10.1186/s12936-017-1963-0)
Supplement: Supplementary file 3 — Additional file 3. Forest plots and other direct and indirect comparisons. Direct and indirect comparisons are extracted from the model with inconsistency. [file 12936_2017_1963_MOESM3_ESM.pdf]

Study	Experimental Events	Experimental Total	Control Events	Control Total	OR	95%-CI	W(fixed)	W(random)
Djalle2014_RCA	57	65	52	65	1.78	[0.68; 4.64]	5.7%	15.1%
Dorsey2007_Uganda	86	111	99	105	0.21	[0.08; 0.53]	5.9%	15.2%
Fanello2007_Rwanda	198	249	243	251	0.13	[0.06; 0.28]	8.8%	15.7%
Faye2007_Senegal	160	161	284	289	2.82	[0.33; 24.32]	1.1%	10.4%
Mutabingwa2005_Tanzania	302	507	450	519	0.23	[0.17; 0.31]	54.3%	16.8%
Sowunmi2007_Nigeria	90	91	84	90	6.43	[0.76; 54.52]	1.1%	10.4%
Zongo2007_BurkFaso	149	184	121	188	2.36	[1.47; 3.79]	23.1%	16.5%
**Fixed effect model**		**1368**		**1507**	**0.44**	**[0.35; 0.55]**	**100%**	**--**
**Random effects model**					**0.76**	**[0.25; 2.30]**	**--**	**100%**

Heterogeneity: I-squared=93.7%, tau-squared=1.897, p<0.0001

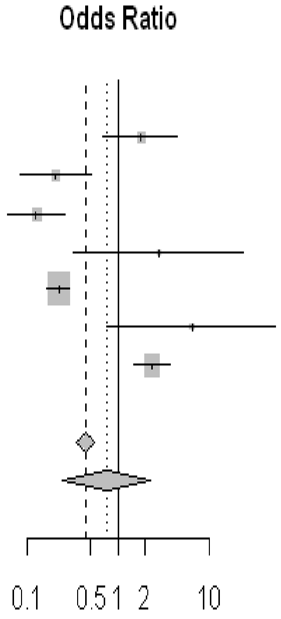

Figure 1: Head-to-head comparison of AQSP=ref and ARLM=control

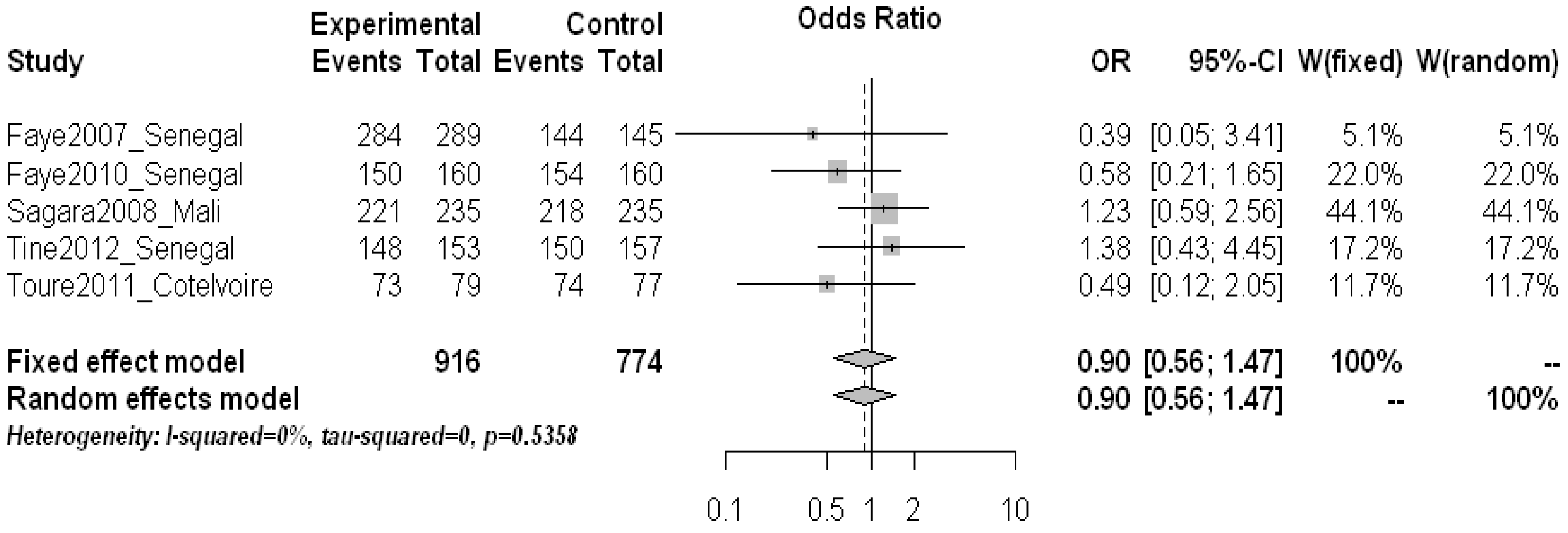

Figure 2: Head-to-head comparison of ARLM=ref and ASMQ=control

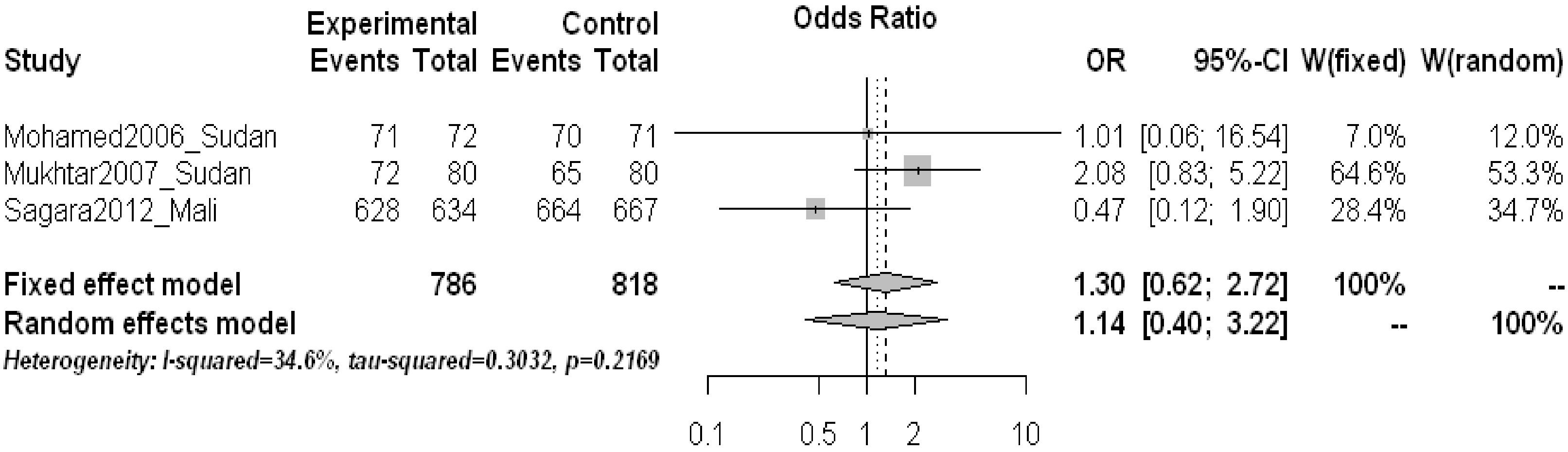

Figure 3: Head-to-head comparison of ARLM=ref and ASSP=control

Study	Experimental Events	Experimental Total	Control Events	Control Total	OR	95%-CI	W(fixed)	W(random)
ABC_Study_group2011	1094	1226	1336	1475	0.86	[0.67; 1.11]	25.0%	7.0%
Adam2010_Sudan	73	74	75	75	0.32	[0.01; 8.09]	0.2%	1.2%
Akindeh2015_Muteng_Garoua	119	144	236	288	1.05	[0.62; 1.77]	5.7%	6.3%
Arinaitwe2009_Uganda	205	320	306	351	0.26	[0.18; 0.39]	10.5%	6.7%
Bassat2009_Afrique_5pays	459	514	938	1039	0.90	[0.64; 1.27]	13.1%	6.8%
Borrmann2011_Kenya_1	64	73	118	149	1.87	[0.84; 4.17]	2.5%	5.4%
Borrmann2011_Kenya_2	150	168	72	84	1.39	[0.64; 3.04]	2.6%	5.5%
Kakuru2014_Uganda_TCC	127	205	149	165	0.17	[0.10; 0.31]	4.6%	6.1%
Men2008_Kenya	62	73	59	73	1.34	[0.56; 3.18]	2.1%	5.2%
Muhindo2014_Uganda	44	96	93	106	0.12	[0.06; 0.24]	3.2%	5.8%
Sawa2013_Kenya	115	153	129	145	0.38	[0.20; 0.71]	3.9%	6.0%
Sow2016_Senegal	120	139	110	124	0.80	[0.38; 1.68]	2.9%	5.7%
Sylla2013_Senegal	176	178	170	176	3.11	[0.62; 15.60]	0.6%	3.1%
Wanzira2014_Uganda	55	158	110	154	0.21	[0.13; 0.34]	6.9%	6.5%
Yavo2011_CMR_SEN_COTI	190	197	180	187	1.06	[0.36; 3.07]	1.4%	4.6%
Yeka2008_Uganda	131	199	186	215	0.30	[0.18; 0.49]	6.6%	6.4%
Yeka2013_Uganda	14	35	54	72	0.22	[0.09; 0.53]	2.1%	5.3%
Zongo2007_BurkFaso	121	188	159	187	0.32	[0.19; 0.52]	6.3%	6.4%
**Fixed effect model**		**4140**		**5065**	**0.53**	**[0.47; 0.60]**	**100%**	**--**
**Random effects model**					**0.53**	**[0.36; 0.77]**	**--**	**100%**

Heterogeneity: I-squared=86.8%, tau-squared=0.5183, p<0.0001

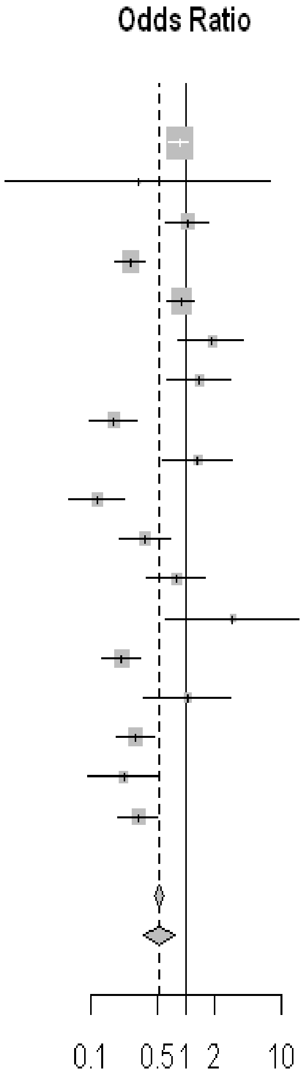

Figure 4: Head-to-head comparison of ARLM=ref and DHPP=control

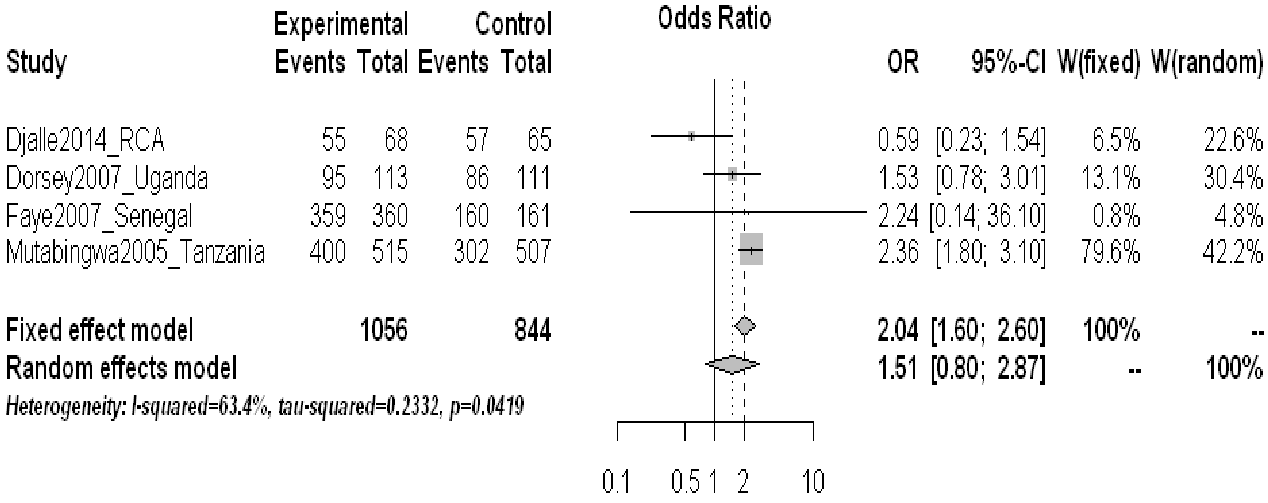

Figure 5: Head-to-head comparison of ASAQ=ref and AQSP=control

Study	Events	Total	Events	Total	OR	95%-CI	W(fixed)	W(random)
ABC_Study_group2011	880	1002	1094	1226	0.87	[0.67; 1.13]	19.7%	6.4%
Adjei2008_Ghana	102	116	97	111	1.05	[0.48; 2.32]	2.2%	3.3%
Akindeh2015_Muteng_Garoua	243	288	119	144	1.13	[0.66; 1.94]	4.7%	4.7%
Ali2013_Cameroon	54	73	62	77	0.69	[0.32; 1.48]	2.3%	3.4%
Burkiwa2006_Uganda	209	211	205	208	1.53	[0.25; 9.25]	0.4%	1.0%
Djalle2014_RCA	55	68	52	65	1.06	[0.45; 2.49]	1.8%	3.0%
Dorsey2007_Uganda	95	113	99	105	0.32	[0.12; 0.84]	1.4%	2.6%
Espie2012_DRC	118	156	106	145	1.14	[0.68; 1.92]	5.0%	4.8%
Falade2008_Nigeria	56	66	59	66	0.66	[0.24; 1.87]	1.3%	2.4%
Falade2014_Nigeria	41	53	46	53	0.52	[0.19; 1.45]	1.3%	2.4%
Faye2007_Senegal	359	360	284	289	6.32	[0.73; 54.40]	0.3%	0.7%
Faye2010_SEN_COTIV	155	159	158	163	1.23	[0.32; 4.65]	0.8%	1.7%
Faye2012_CMR_SEN_CI	243	249	220	232	2.21	[0.82; 5.99]	1.4%	2.5%
Guthmann2006_Angola	61	63	60	64	2.03	[0.36; 11.52]	0.4%	1.1%
Kabanywanyi2007_Tanzanie	48	76	86	99	0.26	[0.12; 0.55]	2.4%	3.5%
Kobbe2008_Ghana	88	123	95	123	0.74	[0.42; 1.32]	4.1%	4.5%
Martensson2005_Zanzibar	191	208	197	200	0.17	[0.05; 0.59]	0.9%	1.9%
Matensson2008_Tanzanie	188	208	185	200	0.76	[0.38; 1.53]	2.8%	3.8%
Meremikwu2006_Nigeria	47	60	47	60	1.00	[0.42; 2.38]	1.8%	3.0%
Mutabingwa2005_Tanzania	457	515	450	519	1.21	[0.83; 1.75]	9.7%	5.8%
Ndiaye2009_Afrique	594	629	297	312	0.86	[0.46; 1.59]	3.5%	4.2%
Ndiaye2011_Senegal	180	184	175	182	1.80	[0.52; 6.26]	0.9%	1.9%
Nhama2014_Mozambique	232	261	335	439	2.48	[1.59; 3.87]	6.8%	5.3%
Owusi_Agyei2008_Ghana	205	220	192	223	2.21	[1.16; 4.21]	3.2%	4.1%
Sagara2012_Mali	593	601	628	634	0.71	[0.24; 2.05]	1.2%	2.3%
Sondo2015_BurkinaFaso	275	340	259	340	1.32	[0.92; 1.91]	10.0%	5.8%
Sow2016_Senegal	110	130	120	139	0.87	[0.44; 1.72]	2.9%	3.9%
Sylla2013_Senegal	175	180	176	178	0.40	[0.08; 2.08]	0.5%	1.2%
Tinto2014_BurkFaso	145	170	148	170	0.86	[0.47; 1.60]	3.5%	4.2%
Yavo2015_CoteIvoire	141	143	144	146	0.98	[0.14; 7.05]	0.3%	0.9%
Yeka2014_Uganda	192	208	194	208	0.87	[0.41; 1.82]	2.4%	3.6%
**Fixed effect model**		**7233**		**7120**	**1.02**	**[0.90; 1.14]**	**100%**	**--**
**Random effects model**					**0.96**	**[0.79; 1.17]**	**--**	**100%**

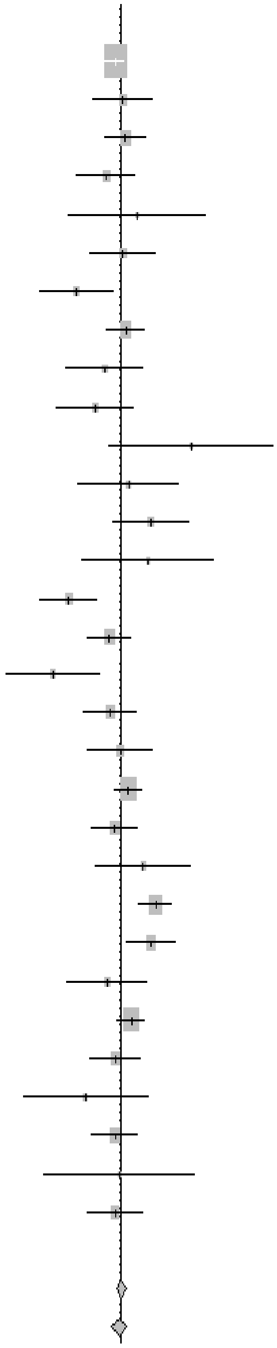

Figure 6: Head-to-head comparison of ASAQ=ref and ARLM=control

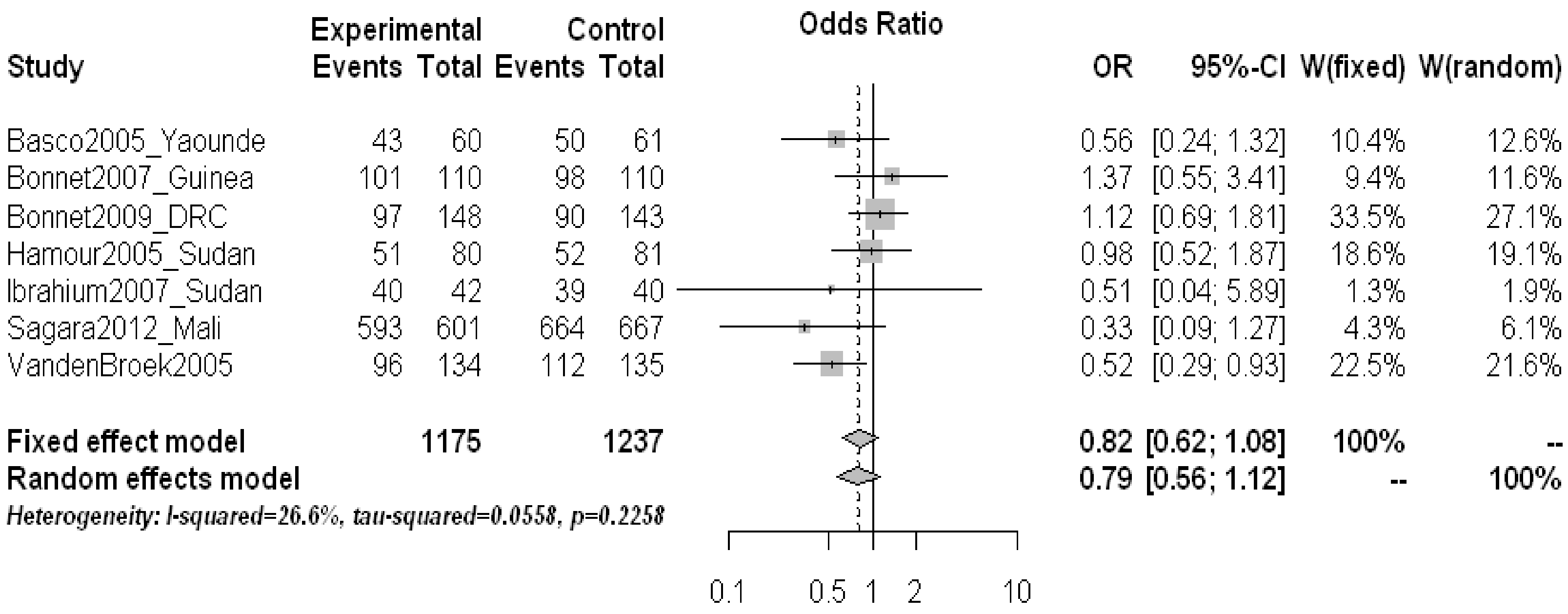

Figure 7: Head-to-head comparison of ASAQ=ref and ASSP=control

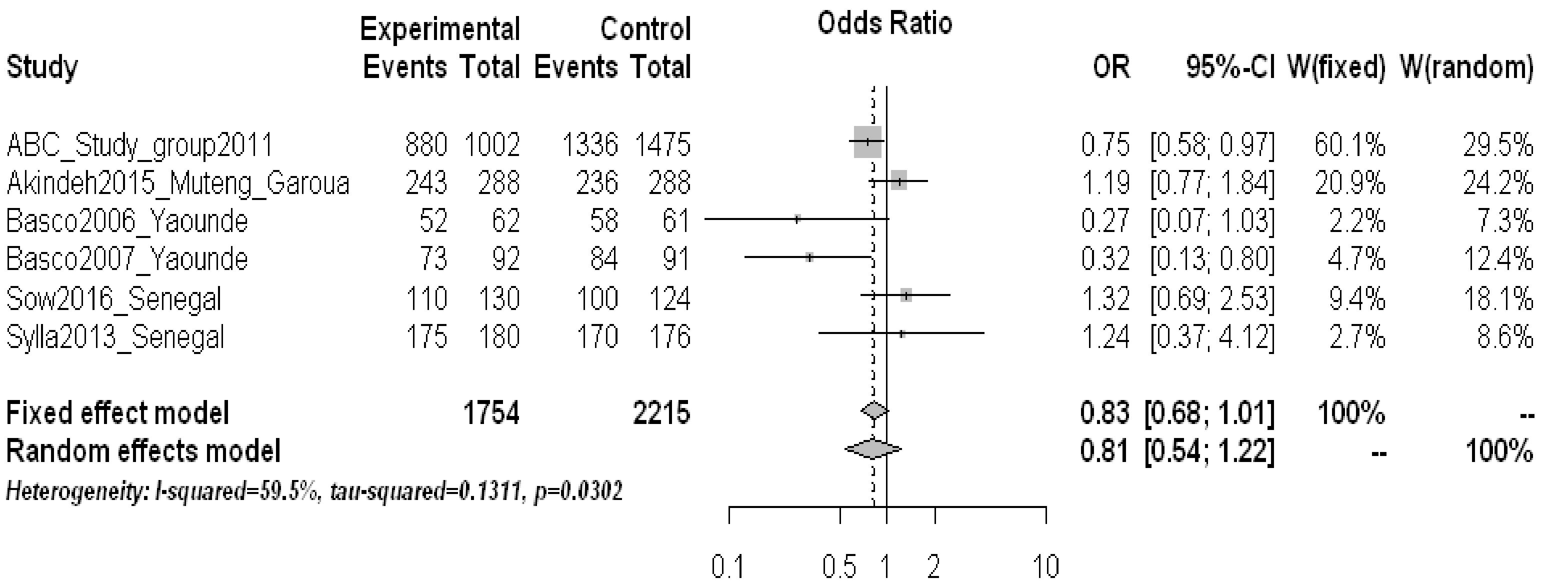

Figure 8: Head-to-head comparison of ASAQ=ref and DHPP=control

Node d[k,k']	Treatment k= control	Treatment k'= reference	Number of trials	Mean LogOR of ref vs ctrl	sd
d[1,2]	ARLM	AQSP	7	-0.0454	0.3923
d[1,3]	ARLM	ASAQ	31	0.08636	0.1855
d[1,4]	ARLM	ASAQCPH	1	0.6474	1.058
d[1,5]	ARLM	ASATPG	0	-0.1407	100.4
d[1,6]	ARLM	ASCD	4	-0.2814	0.5437
d[1,7]	ARLM	ASMQ	5	0.2315	0.5016
d[1,8]	ARLM	ASNAPH	2	-0.1993	1.366
d[1,9]	ARLM	ASPY	2	0.3429	0.6722
d[1,10]	ARLM	ASSMP	3	0.5023	0.6382
d[1,11]	ARLM	ASSP	3	-0.0148	0.6949
d[1,12]	ARLM	DHPP	18	0.6312	0.2367
d[1,13]	ARLM	DHPPT	1	0.3795	1.035
d[2,3]	AQSP	ASAQ	4	-0.0997	100.3
d[2,4]	AQSP	ASAQCPH	0	0.194	100.6
d[2,5]	AQSP	ASATPG	0	0.0818	99.58
d[2,6]	AQSP	ASCD	0	0.2918	99.79
d[2,7]	AQSP	ASMQ	2	0.3891	1.037
d[2,8]	AQSP	ASNAPH	0	-0.1155	99.81
d[2,9]	AQSP	ASPY	0	0.5803	100.2
d[2,10]	AQSP	ASSMP	1	0.3003	99.73
d[2,11]	AQSP	ASSP	1	-0.177	0.9994
d[2,12]	AQSP	DHPP	3	0.7597	0.6557
d[2,13]	AQSP	DHPPT	0	0.7231	100.2
d[3,4]	ASAQ	ASAQCPH	1	0.8938	99.85
d[3,5]	ASAQ	ASATPG	0	1.225	1.086
d[3,6]	ASAQ	ASCD	2	0.6311	99.84
d[3,7]	ASAQ	ASMQ	1	-0.7518	100.1
d[3,8]	ASAQ	ASNAPH	1	-0.1846	99.78
d[3,9]	ASAQ	ASPY	0	0.1836	99.59
d[3,10]	ASAQ	ASSMP	2	-0.6735	0.972
d[3,11]	ASAQ	ASSP	7	0.1922	0.4338
d[3,12]	ASAQ	DHPP	6	1.273	0.7718
d[3,13]	ASAQ	DHPPT	0	0.2256	99.59
d[4,5]	ASAQCPH	ASATPG	0	0.3247	100.1
d[4,6]	ASAQCPH	ASCD	0	0.1505	100.5
d[4,7]	ASAQCPH	ASMQ	0	-0.0691	99.37
d[4,8]	ASAQCPH	ASNAPH	0	0.3129	99.7
d[4,9]	ASAQCPH	ASPY	0	0.2768	99.44
d[4,10]	ASAQCPH	ASSMP	0	0.03252	99.26
d[4,11]	ASAQCPH	ASSP	0	0.659	100.3
d[4,12]	ASAQCPH	DHPP	0	-0.6399	100
d[4,13]	ASAQCPH	DHPPT	0	0.4048	99.95

Table 1: The fitted model with inconsistency assumption provided 2-by-2 comparisons; comparisons given the other treatments as common comparators follow.

Node d[k,k']	Treatment k= control	Treatment k'= reference	Number of trials	Mean LogOR of ref vs ctrl	sd
d[5,6]	ASATPG	ASCD	0	0.173	99.86
d[5,7]	ASATPG	ASMQ	0	-0.0829	99.86
d[5,8]	ASATPG	ASNAPH	0	-0.1185	100.8
d[5,9]	ASATPG	ASPY	0	-1.053	100.1
d[5,10]	ASATPG	ASSMP	0	-0.2764	99.54
d[5,11]	ASATPG	ASSP	0	-0.1506	100.1
d[5,12]	ASATPG	DHPP	0	0.8972	99.79
d[5,13]	ASATPG	DHPPT	0	-0.5034	99.9
d[6,7]	ASCD	ASMQ	0	0.1101	99.93
d[6,8]	ASCD	ASNAPH	0	-0.1616	99.86
d[6,9]	ASCD	ASPY	0	0.02656	99.47
d[6,10]	ASCD	ASSMP	0	0.2459	100
d[6,11]	ASCD	ASSP	1	1.808	1.013
d[6,12]	ASCD	DHPP	1	0.237	100.3
d[6,13]	ASCD	DHPPT	0	-0.0264	100.6
d[7,8]	ASMQ	ASNAPH	0	-0.3731	100.1
d[7,9]	ASMQ	ASPY	0	-0.0517	99.88
d[7,10]	ASMQ	ASSMP	0	-0.2189	99.84
d[7,11]	ASMQ	ASSP	0	-0.049	100.7
d[7,12]	ASMQ	DHPP	0	0.4478	100.2
d[7,13]	ASMQ	DHPPT	0	0.8446	99.29
d[8,9]	ASNAPH	ASPY	0	0.1556	100.2
d[8,10]	ASNAPH	ASSMP	0	-0.5263	100.5
d[8,11]	ASNAPH	ASSP	0	0.3574	99.81
d[8,12]	ASNAPH	DHPP	0	-0.8014	100.2
d[8,13]	ASNAPH	DHPPT	0	-0.4107	99.82
d[9,10]	ASPY	ASSMP	0	0.1542	99.22
d[9,11]	ASPY	ASSP	0	0.04094	99.97
d[9,12]	ASPY	DHPP	0	0.2887	100.2
d[9,13]	ASPY	DHPPT	0	0.2626	100.3
d[10,11]	ASSMP	ASSP	0	-1.56	10 0.4
d[10,12]	ASSMP	DHPP	0	-0.5191	100.3
d[10,13]	ASSMP	DHPPT	0	-0.6216	100
d[11,12]	ASSP	DHPP	0	-0.4688	100.2
d[11,13]	ASSP	DHPPT	0	0.01358	100.7
d[12,13]	DHPP	DHPPT	0	-0.5988	99.78

Table 2: The fitted model with inconsistency assumption provided 2-by-2 comparisons; comparisons given the other treatments as common comparators follow.
